# A Hand Book of the Theory and Practice of Medicine

**Published:** 1874-02

**Authors:** 


### Books Reviewed.

*A Hand Book of the Theory and Practice of Medicine.* By Frederick T. Roberts, M. B., B. Sc., M.R.C.P. Philadelphia: Lindsay & Blakiston, 1874.

Dr. Roberts has succeeded admirabl, in the work which he has undertaken. His book is not intended as a treaties upon the principles and practice of medicine, but rather as a text-book which shall present in a condensed and at the same time useful form the salient points upon the subject. The arrangement of the work is excellent, and the several subj cts are treated in an intelligent manner. The author has departed from the plan usual'y pursued by writers upon medicine. 1st Bf fore describing the individual diseases of the several organs, an outline has been given of the clinical phenomena which indicate a morbid condition of each, and the modes of examination employed in their investigation, while the principal symptoms are considered in detail. 2d. An endeavor has also been made to generalize the remarks upon diagnosis, prognosis and treatment as for as practicable The plan is somewhat novel, but we think will meet with general approbation. In the treatment of the various subjects, the author has at times omitted to mention several points of more or less importance, but these omissions, although detracting somewhat from the value of the work, are to be excused when we take into consideration the object of the author and the general excellence of his work. A copious index adds to the value of the book, which will be found upon study, to be a valuable addition to the list of Hand-Books for students, and which will also be of no little advantage and instruction to the practitioner.

